# Are We Improving? Update and Critical Appraisal of the Reporting of Decision Process and Quality Measures in Trials Evaluating Patient Decision Aids

**DOI:** 10.1177/0272989X211011120

**Published:** 2021-05-08

**Authors:** Logan Trenaman, Jesse Jansen, Jennifer Blumenthal-Barby, Mirjam Körner, Joanne Lally, Daniel Matlock, Lilisbeth Perestelo-Perez, Mary Ropka, Christine Stirling, Kathrene Valentine, Ha Vo, Celia E. Wills, Richard Thomson, Karen Sepucha

**Affiliations:** University of British Columbia, Vancouver, BC, Canada; Centre for Health Evaluation and Outcome Sciences, Vancouver, Canada; Maastricht University, Maastricht, The Netherlands; Center for Medical Ethics and Health Policy, Baylor College of Medicine, Houston, TX, USA; Institute of Medical Psychology and Medical Sociology, Medical Faculty, Albert-Ludwigs- University, Freiburg, Baden-Wurttemberg, Germany; Population Health Sciences Institute, Baddiley Clark Building, Newcastle University, Newcastle upon Tyne, UK; University of Colorado, Aurora, CO, USA; VA Eastern Colorado Geriatric Research Education and Clinical Center, Denver, CO, USA; Evaluation Unit, Canary Islands Health Service, REDISSEC, Tenerife Spain; University of Virginia, Charlottesville, VA, USA; School of Nursing, University of Tasmania, Hobart, Tasmania, Australia; Harvard Medical School, Boston, MA, USA; Massachusetts General Hospital, Boston, MA, USA; Massachusetts General Hospital, Boston, MA, USA; Ohio State University, Columbus, OH, USA; Population Health Sciences Institute, Baddiley Clark Building, Newcastle University, Newcastle upon Tyne, UK; Harvard Medical School, Boston, MA, USA; Massachusetts General Hospital, Boston, MA, USA

**Keywords:** checklist/standards, decision support techniques, patient-centred care, patient decision aids, shared decision making

## Abstract

**Background:**

In 2014, a systematic review found large gaps in the quality of reporting of measures used in 86 published trials evaluating the effectiveness of patient decision aids (PtDAs). The purpose of this study was to update that review.

**Methods:**

We examined measures of decision making used in 49 randomized controlled trials included in the 2014 and 2017 Cochrane Collaboration systematic review of PtDAs. Data on development of the measures, reliability, validity, responsiveness, precision, interpretability, feasibility, and acceptability were independently abstracted by 2 paired reviewers.

**Results:**

Information from 273 measures was abstracted, and 109 of these covered the core domains of decision processes (*n* = 55) and decision quality including informed choice/knowledge (*n* = 48) and values-choice concordance (*n* = 12). Very few studies reported data on the performance and clinical sensibility of measures, with reliability (23%) and validity (6%) being the most common. Studies using new measures were less likely to include information about their psychometric performance compared with previously published measures.

**Limitations:**

The review was limited to reporting of measures in studies included in the Cochrane review and did not consult prior publications.

**Conclusion:**

There continues to be very little reported about the development or performance of measures used to evaluate the effectiveness of PtDAs in published trials. Minimum reporting standards have been published, and efforts to require investigators to use them are needed.

## Introduction

The International Patient Decision Aid Standards (IPDAS) collaboration recommends that patient decision aids (PtDAs) are evaluated by their impact on 2 core domains: decision process and decision quality.^1^ Decision process refers to the extent to which a PtDA helps patients to recognize that a decision needs to be made; feel informed about the options; be clear about what matters most to them in this decision; discuss goals, concerns, and preferences with their health care providers; and be involved in decision making. Decision quality is the extent to which a patient’s eventual choice is informed and consistent with their values. There are many different measures available for these constructs, with new ones being developed and tailored for specific PtDAs.^2,3^ To understand the impact of PtDAs, it is important that trials report on the psychometric properties of the measures that are used.

Several studies have highlighted issues with reporting of measures for evaluating PtDAs including variability in definitions, methodology, and validity and generally poor reporting of psychometrics and development.^4–7^ A 2014 systematic review conducted by several of the authors examined measures used in 86 randomized trials included in the 2011 Cochrane systematic review of PtDAs and found that few provided details on psychometric properties of the individual measures.^8^ This work informed the subsequent development and publication of reporting guidelines for evaluations of PtDAs, the SUNDAE checklist.^9^

This article updates and extends that previous work by conducting a review of the measures used to evaluate decision making in the new trials added to the 2014 and 2017 Cochrane systematic reviews of PtDAs.^10,11^ We focus on the quality of reporting on the development and performance of the outcomes related to decision process and decision quality as recommended by IPDAS.

## Methods

This study updates the previous review and follows a similar approach.^8^ Pairs of reviewers independently reviewed the full-text manuscripts of the 49 new randomized controlled trials included in the 2014 and 2017 Cochrane systematic reviews of PtDAs,^10,11^ determined whether they measured 1 or more of the elements of the “quality of the decision-making process” or “decision quality,” and abstracted information using standard forms. The reviewers collected information on study context, description of the measure(s) and their administration, the development process (item generation, cognitive testing, pilot studies), psychometric performance (reliability, validity, responsiveness), and clinical sensibility (interpretability, feasibility, and acceptability). Table 1 includes some of the abstracted data fields and provides examples of evidence from our past review.^8^ The supplemental file includes details on the studies included in this review and the full data extraction tool.

**Table 1:** Elements Abstracted Regarding Measure Development and Psychometric Performance^a^

Measure Development		Examples of Evidence from Past Review
Item generation	How were content items developed and by whom?	Showing item generation, pilot study, reliability, validity and interpretability: “First, we determined whether subjects were better informed through a twenty question test of BPH knowledge . . . developed by a panel including a general internist, a urologist, a survey researcher, and a lawyer with a special interest in informed consent. Correct responses were scored +1, incorrect responses −1, and “not sure” responses were scored 0 (total range −20 to +20). Validation of new outcome measures Cronbach’s alpha statistic for the items testing BPH knowledge was 0.68. The criterion validity of this test was assessed by comparing scores for a convenience sample of 12 urologic nurses with the scores of the 167 BPH patients enrolled in the baseline period. The nurses had a mean score of 14.8 [out of 20], compared to 5.6 for the patients (p < 0.001). Nurses answered an average of 85% of the questions correctly, compared to 48% for the patients (p < 0.001). Furthermore, a modest correlation between these patients’ knowledge scores and their educational levels was seen, r = 0.23 (p < 0.001).”^12^
Cognitive testing	Was the measure tested for understandability before use?	
Pilot studies	Were pilot studies (of any type) conducted to pre-test the measure?	
Measure performance		
Reliability^b^	Were appropriate assessments of the reliability of the measure reported? If so, was there evidence of adequate reliability?	Showing reliability, validity and responsiveness: “The decisional conflict scale measured patients’ uncertainty about which therapy to choose, modifiable factors contributing to uncertainty (believing themselves to be uninformed, unclear about values, and unsupported in decision making), and perceived effective decision making. The scale is reliable, discriminates between those who make or delay decisions, is responsive to change, and discriminates between different decision-supporting interventions. Two items were added to elicit patients’ perceptions that they were informed about the benefits and risks of warfarin and, separately, about benefits and risks of aspirin. This did not affect the scale’s reliability in this study (Cronbach α=.92).”^13^
Validity^c^	Were appropriate assessments of the validity of the measure reported? If so, was there evidence of adequate validity?	
Responsiveness	Is there evidence that the measure is sensitive to changes of importance to patients and clinicians?	
Clinical sensibility		
Interpretability	Are the scores meaningful to clinicians and patients?	Showing interpretability: A score of 25 out of 100 is “associated with implementing decisions,” and a score of 37.5 out of 100 is “associated with decision delay or feeling unsure about implementation.”^14^
Acceptability^d^	Does the measure appear to be acceptable to respondents?	Showing pilot testing and acceptability: Showing interpretability: “In the literature, assessment of values has primarily been measured with probability-based risk-benefit trade-offs. We pretested these items in focus groups (k = 1; n = 15) and found them unacceptable to a majority of men. Therefore, we developed items to assess the personal importance or relative worth of the advantages and limitations of screening, based on focus groups themes and published literature. Further information about scale development is available elsewhere.”^15^
Feasibility of administration	Are there indicators of the appropriateness of effort, burden, or disruption (of clinical or research team) required to administer and score the measure?	

a Adapted from Sepucha et al.^8^

b Includes internal consistency reliability (e.g., Cronbach’s alpha, Kuder-Richardson coefficient), test-retest reliability, and interrater reliability (e.g., percentage agreement, Kappa coefficient; intraclass correlation coefficient).

c Includes content validity (e.g., Content Validity Index), criterion-related validity (e.g., correlations to demonstrate concurrent, predictive validity), construct validity (e.g., factor analysis to demonstrate predicted convergence/divergence of constructs and/or structural invariance of the measure, discriminant analysis, known groups analysis)

d Could be inferred from patterns of missing data or low response rates.

A measure was considered new if there was no cited prior publication and/or it was not a known, named scale. Articles that cited a reference with respect to any of these issues (e.g. “The Decisional Conflict Scale has been shown to be valid and reliable”^16^) were given credit for reporting those elements. However, we did not consult cited sources to confirm that information or obtain additional unreported information. The abstraction was limited to the details provided within the published trial papers, based on how a reader might evaluate the measures as described by the trial authors. Frequent calls with the entire coding group were held throughout the data abstraction process to ensure consistency. Discrepancies between reviewers were initially discussed by the paired reviewers, and most were resolved after discussion. The lead authors (K.S. and R.T.) adjudicated unresolved disagreements. The data abstracted from the studies are available from the corresponding author by request.

### Analysis

We classified the measures and assessed the presence of reporting for key elements of measure development, psychometric performance, and clinical sensibility. We examined reporting for measures of knowledge, values-choice concordance and decision process. We did not separate out subelements of the decision process (e.g., feel informed), as most measures included multiple elements and did not report separately.

## Results

Of the 49 new trials, 44 (90%) measured at least 1 aspect of decision quality or decision process. Most studies included 1 or more measures of the decision process (78%, 38/49 studies) and knowledge (73%, 36/49 studies), whereas only a minority measured values-choice concordance (24%, 12/49 studies).

We abstracted 273 reported measures related to decision making. Of these, 109 covered 1 or more core constructs of the decision process (*n* = 55) or decision quality, including knowledge (*n* = 48) or values-choice concordance (*n* = 12; Table 2). Of note, 6 measures covered both knowledge and concordance. The most common other type of outcomes included actual choice (*n* = 40), preference or preferred choice (*n* = 25), satisfaction with decision making or chosen option (*n* = 17), depression and/or anxiety (*n* = 14), adherence (*n* = 8), and decision regret (*n* = 7).

**Table 2:** Reporting on Performance of New and Established Measures of Decision Quality and Decision Process in Studies of PtDAs

	Core Outcomes (*n* = 109)							
			Decision Quality					
	Decision Process (*n* = 55)		Knowledge^a^ (*n* = 48)		Concordance (*n* = 12)		Other Decision Outcomes (*n* = 164)	
	*n*/*N*	%	*n*/*N*	%	*n*/*N*	%	*n*/*N*	%
Previously published	50/55	91	28/48	57	5/12	42	75/164	46
Measure development								
Development process	1/55	2	7/48	15	2/12	17	10/164	6
Item generation	1/55	2	6/48	13	1/12	8	6/164	4^b^
Cognitive testing	0/55	0	4/48	8	0/2	0	4/164	2^b^
Pilot studies	0/55	0	4/48	8	0/2	0	3/164	2^b^
Measure performance								
Reliability	18/55	33	6/48	13	1/12	8	19/164	12
Validity	4/55	7	1/48	2	1/12	8	5/164	2
Responsiveness	1/55	2	0/48	0	0/12	0	0/164	0
Clinical sensibility								
Interpretability	3/55	5	2/48	4	0/12	0	0/164	0
Acceptability	5/55	9	1/48	2	1/12	8	5/164	3
Feasibility of administration	0/55	0	1/48	2	1/12	8	1/164	1

a Six measures covered both knowledge and concordance.

b Missing data on development process for *n* = 3.

Studies included very limited information on psychometric properties of the measures (*n* = 109) such as reliability (23%), validity (6%), and responsiveness (1%). Studies rarely assessed the clinical sensibility of the measures, such as feasibility (2%), acceptability (7%), and interpretability (5%).

Whereas most decision process measures used and cited a previously published measure (50/55), many knowledge (20/48) and concordance measures (7/12) did not cite an existing measure. Few studies using new measures provided information on the development process (4/31, 13%) or psychometric properties (6/31, 19%). Previously published measures were significantly more likely to have some reporting of psychometrics (41% v. 19%, χ^2^ = 0.04).

## Discussion

Decision process and quality measures are critical to evaluating the effectiveness of PtDAs.^1^ This brief report updates a previous review,^8^ summarizing new evidence on the quality of reporting of measures of decision process and quality captured by 49 new studies included in the 2014 and 2017 Cochrane Collaboration’s reviews of PtDAs. This review finds continued shortcomings in the reporting of the development, performance, and clinical sensibility of decision process and quality measures used in published trials.

Reporting of the development process for new measures was poor. Generally speaking, previously published measures were more likely to have some reporting of psychometrics than new measures (41% v. 19%); however, this largely reflects strong reporting of the Decision Conflict Scale (DCS).^17^ The DCS was used in more than half of the trials (72/135, 53%), often with detailed descriptions of performance.

Most new trials include decision-making evaluation measures (90%), which is similar to the previous review (88%).^8^ Reliability reporting was also similar (23% v. 21%), whereas validity was worse (6% v. 16%) in these new studies. A common misperception is that validity and reliability are properties of the survey instrument, when in reality they are properties of data and the interpretation of the data (which includes understanding the administration, setting, sample, and analysis procedures).^18^ This underscores the importance of reporting relevant information on psychometric performance for each study and each use of an instrument or measure. Detailed reporting of psychometric properties is important to allow appropriate interpretation of results, improve our understanding of the impact of PtDAs on decision process and outcomes, and support replication and synthesis of findings.^19^ There are many great resources that describe how to assess the adequacy of psychometric evidence, with the authors recommending a text by Waltz et al.^20^ The SUNDAE checklist was developed in 2018 to support completeness and transparency of reporting of PtDA evaluation studies, including psychometric properties of the measures used.^9^ While the checklist did not affect this update, which included trials published up to 2017, it may improve reporting in future, particularly if journals adopt the SUNDAE checklist.

Few studies include details on the clinical sensibility of the measures. This information is important to allow appropriate interpretation of the results and to support successful implementation of trials into routine clinical practice. Patient-reported measures provide insight into the outcomes and experience of care from the patients’ perspective and are valuable to monitor quality of care and outcomes.^21–24^ However, without information on the acceptability, feasibility, and interpretability of the measures, their implementation into practice may be hindered.

Our study has several limitations. First, we focused on randomized controlled trials included within the Cochrane review, although we would expect these to be the highest-quality evaluations. Second, we did not review the cited sources of previously published measures; hence, our findings reflect only the quality of the reporting of measures not the quality of the measures themselves. Third, it is possible that developers reported more details about the measures elsewhere, and this would not have been captured in our review.

Several questions remain to be answered. What other measures should be used to evaluate PtDAs, if any (e.g., health outcomes, cost-effectiveness, potential harms), and when should they be measured? What components of PtDAs are core to effectiveness? Are different measures needed for disadvantaged patients (e.g., individuals with low literacy or low incomes)? Increasingly, 1 or more options in situations covered in PtDAs involve a large behavior change component (e.g., surgery versus diet and exercise for obesity/weight management). In what ways does this behavior change component change our strategies (if at all) for the evaluation of PtDAs (e.g., do we need to assess levels of self-efficacy and motivation in addition to knowledge and concordance)? How do we support decisions in which an option is considered of low value (e.g., prostate-specific antigen screening for certain groups)?

There are also theoretical issues. A growing body of research suggests that defining what a good medical decision is, and how to measure it, is more complicated than is often assumed in theoretical decision-making frameworks.^25^ For example, real-life decision making is influenced by interpersonal factors, structural constraints, and affect/emotions. It provides an argument for consideration of how these factors (and others) contribute to the definition of good medical decision making and a tailored approach to the measurement of decision quality.

There continues to be very little reported about the development or performance of measures used to evaluate the effectiveness of PtDAs within published trials. Minimum reporting standards (SUNDAE) have been published, and wide use should be promoted to support transparent and accurate reporting and clearer interpretation of the outcomes of PtDA trials.

## Supplemental Material

sj-docx-1-mdm-10.1177_0272989X211011120 – Supplemental material for Are We Improving? Update and Critical Appraisal of the Reporting of Decision Process and Quality Measures in Trials Evaluating Patient Decision Aids Click here for additional data file. Supplemental material, sj-docx-1-mdm-10.1177_0272989X211011120 for Are We Improving? Update and Critical Appraisal of the Reporting of Decision Process and Quality Measures in Trials Evaluating Patient Decision Aids by Logan Trenaman, Jesse Jansen, Jennifer Blumenthal-Barby, Mirjam Körner, Joanne Lally, Daniel D. Matlock, Lilisbeth Perestelo-Perez, Mary Ropka, Christine Stirling, Kathrene Valentine, Ha Vo, Celia E. Wills, Richard Thomson and Karen Sepucha in Medical Decision Making
