# Supplementary material for: Are We Improving? Update and Critical Appraisal of the Reporting of Decision Process and Quality Measures in Trials Evaluating Patient Decision Aids
Source: Med Decis Making. 2021 May 8;41(7):954–9. doi: 10.1177/0272989X211011120 (PMC8474325; doi:10.1177/0272989X211011120)
Supplement: sj-docx-1-mdm-10.1177_0272989X211011120 – Supplemental material for Are We Improving? Update and Critical Appraisal of the Reporting of Decision Process and Quality Measures in Trials Evaluating Patient Decision Aids [file sj-docx-1-mdm-10.1177_0272989X211011120.docx]

**Table of included studies and outcomes**

| **Ref #** | **First author** | **Date** | **Topic** | **Decision Quality Outcomes** | **Decision Process Outcomes** | **Other Outcomes** |
| --- | --- | --- | --- | --- | --- | --- |
| 1 | Allen | 2010 | Prostate cancer screening | Knowledge  Concordance | DCS  CPS | Decision self-efficacy  Stage of decision making |
| 2 | Arterburn | 2011 | Bariatric surgery | Knowledge  Concordance | DCS | Decision self-efficacy  Preference  Preferred choice  Outcomes expectancies |
| 3 | Berry | 2013 | Prostate cancer |  | DCS | Choice  Acceptability  Anxiety |
| 4 | Bjorklund | 2012 | Down Syndrome Screening | MMIC  Knowledge | Experience with Information | Choice  Attitude |
| 5 | Bozic | 2013 | Knee osteoarthritis | Knowledge |  | Choice  Satisfaction  Stage of Decision making |
| 6 | Brazell | 2014 | Pelvic organ prolapse |  | DCS | Choice  Decision Regret |
| 7 | Chabrera | 2015 | Prostate cancer | Knowledge | DCS | Satisfaction  Coping |
| 8 | Chambers | 2012 | Influenza immunization |  |  | Choice  Confidence |
| 9 | de Achaval | 2012 | Knee osteoarthritis |  | DCS | Preferred choice |
| 10 | Evans | 2010 | Prostate cancer screening | Knowledge | DCS | Choice  Intention  Preference  Anxiety |
| 11 | Fagerlin | 2011 | Breast cancer prevention |  | DCS | Choice  Intention  Preference  Risk perception |
| 12 | Fraenkel | 2012 | Nonvalvular atrial fibrillation | Knowledge  Realistic expectations | DCS | Choice  Preference  Anxiety  Discussion with Doctor |
| 13 | Hanson | 2011 | Assisted feeding options | Knowledge  Realistic expectations | DCS | Choice  Decision regret  Satisfaction  Discussion with doctor |
| 14 | Hess | 2012 | Cardiac stress testing | Knowledge | DCS  OPTION | Choice  Satisfaction  Trust in Physician |
| 15 | Jibaja-Weiss | 2011 | Breast cancer surgery | Knowledge | DCS | Choice  Satisfaction (2) |
| 16 | Knops | 2014 | Abdominal aortic aneurism | Knowledge | DCS | Choice  Anxiety/Depression  Quality of life |
| 17 | Kupke | 2013 | Dental treatment | Knowledge |  | Preference  Satisfaction |
| 18 | Kuppermann | 2014 | Prenatal genetic testing | Knowledge  Realistic expectations | DCS | Choice  Decision Regret |
| 19 | Labrecque | 2010 | Vasectomy | Knowledge | DCS | Preferred choice |
| 20 | Lam | 2013 | Breast cancer surgery | Knowledge | DCS  OPTION  Perceived decision making difficulties | Choice  Decision Regret  Anxiety/Depression  Quality of life |
| 21 | Langston | 2010 | Contraception |  |  | Choice  Adherence |
| 22 | LeBlanc | 2015 | Osteoporosis | Knowledge  Realistic expectations | DCS  OPTION | Choice  Adherence  Clinician DCS  Satisfaction  QoL  Duration of encounter |
| 23 | Legare | 2012 | Antiobiotics for acute respiratory infection |  | DCS  CPS  Perceived decision quality | Choice  Intention  Decision regret |
| 24 | Legare | 2011 | Antiobiotics for acute respiratory infection |  | DCS  CPS  Perceived decision quality | Adherence  Intention  Decision regret  Desire for shared decision making |
| 25 | Leighl | 2011 | Colorectal cancer chemotherapy | Knowledge | DCS  CPS | Choice  Preference  Satisfaction with decision aid  Satisfaction with visit  Information style  Anxiety |
| 26 | Lepore | 2012 | Prostate cancer screening | Knowledge  Concordance | DCS | Choice  Preference  Anxiety/Depression |
| 27 | Mann | 2010 | High cholesterol | Knowledge  Realistic expectation | DCS | Adherence  Beliefs about medicines |
| 28 | Marteau | 2010 | Diabetes screening |  |  | Choice  Intention  Satisfaction |
| 29 | Mathers | 2012 | Diabetes | Knowledge | DCS  CPS | Preferred choice  Perceived risk  Decision regret |
| 30 | Mathieu | 2010 | Breast cancer screening | MMIC  Knowledge |  | Choice  Attitudes |
| 31 | McCaffery | 2010 | Cervical cancer screening | Knowledge |  | Risk perception  Satisfaction (2)  Cancer screening questionnaire  Anxiety  Worry  Self esteem  Intrusive thoughts |
| 32 | Miller | 2011 | Colorectal cancer screening |  | Stage of change | Choice  Preferred choice |
| 33 | Montori | 2011 | Osteoporosis | Knowledge | DCS  OPTION | Choice  Adherence (2)  Satisfaction  Trust in physician |
| 34 | Mott | 2014 | Mental health |  |  | Choice  Preference |
| 35 | Myers | 2011 | Prostate cancer screening | Knowledge | DCS  Braddock Informed Choice | Choice  Preference |
| 36 | Raynes-Greenow | 2010 | Childbirth | Knowledge  Concordance | DCS  CPS | Choice  Satisfaction  Stage of decision making  Anxiety |
| 37 | Rubel | 2010 | Prostate cancer screening | Knowledge | DCS  CPS | Preference (2)  Risk perception  Anxiety |
| 38 | Sawka | 2012 | Thyroid cancer | Knowledge | DCS  CPS | Choice  Preferred choice |
| 39 | Schroy | 2011 | Colorectal cancer screening | Knowledge  Concordance |  | Choice  Preference (2)  Satisfaction |
| 40 | Schwalm | 2012 | Coronary angiogram access | Knowledge  Concordance | DCS | Choice  Risk perception |
| 41 | Sheridan | 2011 | Heart disease |  |  | Choice  Adherence |
| 42 | Shourie | 2013 | MMR Vaccine | Knowledge | DCS | Choice  Cognitions  Beliefs  Anxiety  Engagement with decision aid |
| 43 | Smith | 2010 | Colorectal cancer screening | MMIC  Knowledge | DCS  CPS | Choice  Preference  Decision self-efficacy  Anxiety  Worry |
| 44 | Solberg | 2010 | Uterine fibroids | Knowledge  Concordance | Treatments offered | Choice  Preference  Satisfaction |
| 45 | Stacey | 2014 | Knee Osteoarthritis | Knowledge  Concordance | SURE scale  Preparation for decision making scale | Choice |
| 46 | Steckelberg | 2011 | Colorectal cancer screening | MMIC  Knowledge |  | Choice  Preference |
| 47 | Taylor | 2006 | Prostate cancer screening | Knowledge | DCS | Choice  Intention |
| 48 | van Peperstraten | 2010 | Fertility | Knowledge | Decision Evaluation Scale  Perceived Knowledge | Choice  Self-efficacy  Anxiety  Depression  Cost |
| 49 | Williams | 2013 | Prostate cancer screening |  | DCS | Choice  Satisfaction  Discussion with doctor |

DCS=Decisional Conflict Scale; CPS=Control Preference Scale; MMIC=Multidimensional Measure of Informed Consent

**References:**

1. Allen JD, Othus MK, Hart A Jr, Tom L, Li Y, Berry D, et al. A randomized trial of a computer-tailored decision aid to improve prostate cancer screening decisions: results from the Take the Wheel trial. Cancer Epidemiol Biomarkers Prev. 2010 Sep;19(9):2172-86. doi: 10.1158/1055-9965.EPI-09-0410. Epub 2010 Aug 17. PMID: 20716619; PMCID: PMC4386842.
2. Arterburn DE, Westbrook EO, Bogart TA, Sepucha KR, Bock SN, Weppner WG. Randomized trial of a video-based patient decision aid for bariatric surgery. Obesity (Silver Spring). 2011 Aug;19(8):1669-75. doi: 10.1038/oby.2011.65. Epub 2011 Apr 7. PMID: 21475138.
3. Berry DL, Halpenny B, Hong F, Wolpin S, Lober WB, Russell KJ, et al. The Personal Patient Profile-Prostate decision support for men with localized prostate cancer: a multi-center randomized trial. Urol Oncol. 2013 Oct;31(7):1012-21. doi: 10.1016/j.urolonc.2011.10.004. Epub 2011 Dec 7. PMID: 22153756; PMCID: PMC3349002.
4. Björklund U, Marsk A, Levin C, Öhman SG. Audiovisual information affects informed choice and experience of information in antenatal Down syndrome screening--a randomized controlled trial. Patient Educ Couns. 2012 Mar;86(3):390-5. doi: 10.1016/j.pec.2011.07.004. Epub 2011 Jul 31. PMID: 21807474.
5. Bozic KJ, Belkora J, Chan V, Youm J, Zhou T, Dupaix J, et al. Shared decision making in patients with osteoarthritis of the hip and knee: results of a randomized controlled trial. J Bone Joint Surg Am. 2013 Sep 18;95(18):1633-9. doi: 10.2106/JBJS.M.00004. PMID: 24048550.
6. Brazell HD, O'Sullivan DM, Forrest A, Greene JF. Effect of a Decision Aid on Decision Making for the Treatment of Pelvic Organ Prolapse. Female Pelvic Med Reconstr Surg. 2015 Jul-Aug;21(4):231-5. doi: 10.1097/SPV.0000000000000149. PMID: 25521472.
7. Chabrera C, Zabalegui A, Bonet M, Caro M, Areal J, González JR, et al. A Decision Aid to Support Informed Choices for Patients Recently Diagnosed With Prostate Cancer: A Randomized Controlled Trial. Cancer Nurs. 2015 May-Jun;38(3):E42-50. doi: 10.1097/NCC.0000000000000170. PMID: 25010250.
8. Chambers LW, Wilson K, Hawken S, Puxty J, Crowe L, Lam PP, et al. Impact of the Ottawa Influenza Decision Aid on healthcare personnel's influenza immunization decision: a randomized trial. J Hosp Infect. 2012 Nov;82(3):194-202. doi: 10.1016/j.jhin.2012.08.003. Epub 2012 Sep 24. PMID: 23017384.
9. de Achaval S, Fraenkel L, Volk RJ, Cox V, Suarez-Almazor ME. Impact of educational and patient decision aids on decisional conflict associated with total knee arthroplasty. Arthritis Care Res (Hoboken). 2012 Feb;64(2):229-37. doi: 10.1002/acr.20646. PMID: 21954198; PMCID: PMC3634330.
10. Evans R, Joseph-Williams N, Edwards A, Newcombe RG, Wright P, Kinnersley P, et al. Supporting informed decision making for prostate specific antigen (PSA) testing on the web: an online randomized controlled trial. J Med Internet Res. 2010 Aug 6;12(3):e27. doi: 10.2196/jmir.1305. PMID: 20693148; PMCID: PMC2956331.
11. Fagerlin A, Dillard AJ, Smith DM, Zikmund-Fisher BJ, Pitsch R, McClure JB, et al. Women's interest in taking tamoxifen and raloxifene for breast cancer prevention: response to a tailored decision aid. Breast Cancer Res Treat. 2011 Jun;127(3):681-8. doi: 10.1007/s10549-011-1450-1. Epub 2011 Mar 26. PMID: 21442198; PMCID: PMC3742062.
12. Fraenkel L, Street RL Jr, Towle V, O'Leary JR, Iannone L, Van Ness PH, et al. A pilot randomized controlled trial of a decision support tool to improve the quality of communication and decision-making in individuals with atrial fibrillation. J Am Geriatr Soc. 2012 Aug;60(8):1434-41. doi: 10.1111/j.1532-5415.2012.04080.x. Epub 2012 Aug 2. PMID: 22861171; PMCID: PMC3419306.
13. Hanson LC, Carey TS, Caprio AJ, Lee TJ, Ersek M, Garrett J, et al. Improving decision-making for feeding options in advanced dementia: a randomized, controlled trial. J Am Geriatr Soc. 2011 Nov;59(11):2009-16. doi: 10.1111/j.1532-5415.2011.03629.x. Epub 2011 Sep 15. PMID: 22091750; PMCID: PMC3227016.
14. Hess EP, Knoedler MA, Shah ND, Kline JA, Breslin M, Branda ME, et al. The chest pain choice decision aid: a randomized trial. Circ Cardiovasc Qual Outcomes. 2012 May;5(3):251-9. doi: 10.1161/CIRCOUTCOMES.111.964791. Epub 2012 Apr 10. PMID: 22496116.
15. Jibaja-Weiss ML, Volk RJ, Granchi TS, Neff NE, Robinson EK, Spann SJ, et al. Entertainment education for breast cancer surgery decisions: a randomized trial among patients with low health literacy. Patient Educ Couns. 2011 Jul;84(1):41-8. doi: 10.1016/j.pec.2010.06.009. Epub 2010 Jul 7. PMID: 20609546.
16. Knops AM, Goossens A, Ubbink DT, Balm R, Koelemay MJ, Vahl AC, et al. A decision aid regarding treatment options for patients with an asymptomatic abdominal aortic aneurysm: a randomised clinical trial. Eur J Vasc Endovasc Surg. 2014 Sep;48(3):276-83. doi: 10.1016/j.ejvs.2014.04.016. Epub 2014 Jun 7. PMID: 24913683.
17. Kupke J, Wicht MJ, Stützer H, Derman SH, Lichtenstein NV, Noack MJ. Does the use of a visualised decision board by undergraduate students during shared decision-making enhance patients' knowledge and satisfaction? - A randomised controlled trial. Eur J Dent Educ. 2013 Feb;17(1):19-25. doi: 10.1111/eje.12002. Epub 2012 Sep 14. PMID: 23279388.
18. Kuppermann M, Pena S, Bishop JT, Nakagawa S, Gregorich SE, Sit A, et al. Effect of enhanced information, values clarification, and removal of financial barriers on use of prenatal genetic testing: a randomized clinical trial. JAMA. 2014 Sep 24;312(12):1210-7. doi: 10.1001/jama.2014.11479. PMID: 25247517; PMCID: PMC4445462.
19. Labrecque M, Paunescu C, Plesu I, Stacey D, Légaré F. Evaluation of the effect of a patient decision aid about vasectomy on the decision-making process: a randomized trial. Contraception. 2010 Dec;82(6):556-62. doi: 10.1016/j.contraception.2010.05.003. Epub 2010 Jun 17. PMID: 21074020.
20. Lam WW, Chan M, Or A, Kwong A, Suen D, Fielding R. Reducing treatment decision conflict difficulties in breast cancer surgery: a randomized controlled trial. J Clin Oncol. 2013 Aug 10;31(23):2879-85. doi: 10.1200/JCO.2012.45.1856. Epub 2013 Jul 8. PMID: 23835709.
21. Langston AM, Rosario L, Westhoff CL. Structured contraceptive counseling--a randomized controlled trial. Patient Educ Couns. 2010 Dec;81(3):362-7. doi: 10.1016/j.pec.2010.08.006. PMID: 20869187.
22. LeBlanc A, Wang AT, Wyatt K, Branda ME, Shah ND, Van Houten H, et al. Encounter Decision Aid vs. Clinical Decision Support or Usual Care to Support Patient-Centered Treatment Decisions in Osteoporosis: The Osteoporosis Choice Randomized Trial II. PLoS One. 2015 May 26;10(5):e0128063. doi: 10.1371/journal.pone.0128063. PMID: 26010755; PMCID: PMC4444262.
23. Légaré F, Labrecque M, Cauchon M, Castel J, Turcotte S, Grimshaw J. Training family physicians in shared decision-making to reduce the overuse of antibiotics in acute respiratory infections: a cluster randomized trial. CMAJ. 2012 Sep 18;184(13):E726-34. doi: 10.1503/cmaj.120568. Epub 2012 Jul 30. PMID: 22847969; PMCID: PMC3447039.
24. Légaré F, Labrecque M, LeBlanc A, Njoya M, Laurier C, Côté L, et al. Training family physicians in shared decision making for the use of antibiotics for acute respiratory infections: a pilot clustered randomized controlled trial. Health Expect. 2011 Mar;14 Suppl 1(Suppl 1):96-110. doi: 10.1111/j.1369-7625.2010.00616.x. PMID: 20629764; PMCID: PMC3073122.
25. Leighl NB, Shepherd HL, Butow PN, Clarke SJ, McJannett M, Beale PJ, et al. Supporting treatment decision making in advanced cancer: a randomized trial of a decision aid for patients with advanced colorectal cancer considering chemotherapy. J Clin Oncol. 2011 May 20;29(15):2077-84. doi: 10.1200/JCO.2010.32.0754. Epub 2011 Apr 11. PMID: 21483008.
26. Lepore SJ, Wolf RL, Basch CE, Godfrey M, McGinty E, Shmukler C, et al. Informed decision making about prostate cancer testing in predominantly immigrant black men: a randomized controlled trial. Ann Behav Med. 2012 Dec;44(3):320-30. doi: 10.1007/s12160-012-9392-3. PMID: 22825933; PMCID: PMC3500675.
27. Mann DM, Ponieman D, Montori VM, Arciniega J, McGinn T. The Statin Choice decision aid in primary care: a randomized trial. Patient Educ Couns. 2010 Jul;80(1):138-40. doi: 10.1016/j.pec.2009.10.008. Epub 2009 Dec 2. PMID: 19959322.
28. Marteau TM, Mann E, Prevost AT, Vasconcelos JC, Kellar I, Sanderson S, et al. Impact of an informed choice invitation on uptake of screening for diabetes in primary care (DICISION): randomised trial. BMJ. 2010 May 13;340:c2138. doi: 10.1136/bmj.c2138. PMID: 20466791; PMCID: PMC2869404.
29. Mathers N, Ng CJ, Campbell MJ, Colwell B, Brown I, Bradley A. Clinical effectiveness of a patient decision aid to improve decision quality and glycaemic control in people with diabetes making treatment choices: a cluster randomised controlled trial (PANDAs) in general practice. BMJ Open. 2012 Nov 5;2(6):e001469. doi: 10.1136/bmjopen-2012-001469. PMID: 23129571; PMCID: PMC3532975.
30. Mathieu E, Barratt AL, McGeechan K, Davey HM, Howard K, Houssami N. Helping women make choices about mammography screening: an online randomized trial of a decision aid for 40-year-old women. Patient Educ Couns. 2010 Oct;81(1):63-72. doi: 10.1016/j.pec.2010.01.001. Epub 2010 Feb 10. PMID: 20149953.
31. McCaffery KJ, Irwig L, Turner R, Chan SF, Macaskill P, Lewicka M, et al. Psychosocial outcomes of three triage methods for the management of borderline abnormal cervical smears: an open randomised trial. BMJ. 2010 Feb 23;340:b4491. doi: 10.1136/bmj.b4491. PMID: 20179125; PMCID: PMC2827716.
32. Miller DP Jr, Spangler JG, Case LD, Goff DC Jr, Singh S, Pignone MP. Effectiveness of a web-based colorectal cancer screening patient decision aid: a randomized controlled trial in a mixed-literacy population. Am J Prev Med. 2011 Jun;40(6):608-15. doi: 10.1016/j.amepre.2011.02.019. PMID: 21565651; PMCID: PMC3480321.
33. Montori VM, Shah ND, Pencille LJ, Branda ME, Van Houten HK, Swiglo BA, et al. Use of a decision aid to improve treatment decisions in osteoporosis: the osteoporosis choice randomized trial. Am J Med. 2011 Jun;124(6):549-56. doi: 10.1016/j.amjmed.2011.01.013. PMID: 21605732.
34. Mott JM, Stanley MA, Street RL Jr, Grady RH, Teng EJ. Increasing engagement in evidence-based PTSD treatment through shared decision-making: a pilot study. Mil Med. 2014 Feb;179(2):143-9. doi: 10.7205/MILMED-D-13-00363. PMID: 24491609.
35. Myers RE, Daskalakis C, Kunkel EJ, Cocroft JR, Riggio JM, Capkin M, Braddock CH 3rd. Mediated decision support in prostate cancer screening: a randomized controlled trial of decision counseling. Patient Educ Couns. 2011 May;83(2):240-6. doi: 10.1016/j.pec.2010.06.011. Epub 2010 Jul 8. PMID: 20619576.
36. Raynes-Greenow CH, Nassar N, Torvaldsen S, Trevena L, Roberts CL. Assisting informed decision making for labour analgesia: a randomised controlled trial of a decision aid for labour analgesia versus a pamphlet. BMC Pregnancy Childbirth. 2010 Apr 8;10:15. doi: 10.1186/1471-2393-10-15. PMID: 20377844; PMCID: PMC2868791.
37. Rubel SK, Miller JW, Stephens RL, Xu Y, Scholl LE, Holden EW, et al. Testing the effects of a decision aid for prostate cancer screening. J Health Commun. 2010 Apr;15(3):307-21. doi: 10.1080/10810731003686614. PMID: 20432110.
38. Sawka AM, Straus S, Rotstein L, Brierley JD, Tsang RW, Asa S, et al. Randomized controlled trial of a computerized decision aid on adjuvant radioactive iodine treatment for patients with early-stage papillary thyroid cancer. J Clin Oncol. 2012 Aug 10;30(23):2906-11. doi: 10.1200/JCO.2011.41.2734. Epub 2012 Jul 2. PMID: 22753906.
39. Schroy PC 3rd, Emmons K, Peters E, Glick JT, Robinson PA, Lydotes MA, et al. The impact of a novel computer-based decision aid on shared decision making for colorectal cancer screening: a randomized trial. Med Decis Making. 2011 Jan-Feb;31(1):93-107. doi: 10.1177/0272989X10369007. Epub 2010 May 18. PMID: 20484090; PMCID: PMC4165390.
40. Schwalm JD, Stacey D, Pericak D, Natarajan MK. Radial artery versus femoral artery access options in coronary angiogram procedures: randomized controlled trial of a patient-decision aid. Circ Cardiovasc Qual Outcomes. 2012 May;5(3):260-6. doi: 10.1161/CIRCOUTCOMES.111.962837. Epub 2012 Apr 10. PMID: 22496115.
41. Sheridan SL, Draeger LB, Pignone MP, Keyserling TC, Simpson RJ Jr, Rimer B, et al. A randomized trial of an intervention to improve use and adherence to effective coronary heart disease prevention strategies. BMC Health Serv Res. 2011 Dec 5;11:331. doi: 10.1186/1472-6963-11-331. PMID: 22141447; PMCID: PMC3268742.
42. Shourie S, Jackson C, Cheater FM, Bekker HL, Edlin R, Tubeuf S, et al. A cluster randomised controlled trial of a web based decision aid to support parents' decisions about their child's Measles Mumps and Rubella (MMR) vaccination. Vaccine. 2013 Dec 5;31(50):6003-10. doi: 10.1016/j.vaccine.2013.10.025. Epub 2013 Oct 19. PMID: 24148574; PMCID: PMC3898271.
43. Smith SK, Trevena L, Simpson JM, Barratt A, Nutbeam D, McCaffery KJ. A decision aid to support informed choices about bowel cancer screening among adults with low education: randomised controlled trial. BMJ. 2010 Oct 26;341:c5370. doi: 10.1136/bmj.c5370. PMID: 20978060; PMCID: PMC2965151.
44. Solberg LI, Asche SE, Sepucha K, Thygeson NM, Madden JE, Morrissey L, et al. Informed choice assistance for women making uterine fibroid treatment decisions: a practical clinical trial. Med Decis Making. 2010 Jul-Aug;30(4):444-52. doi: 10.1177/0272989X09353947. Epub 2009 Nov 30. PMID: 19949063.
45. Stacey, D., Hawker, G., Dervin, G. Tugwell P, Boland L, Pomey MP, et al. Decision aid for patients considering total knee arthroplasty with preference report for surgeons: a pilot randomized controlled trial. *BMC Musculoskelet Disord* **15,**54 (2014). [doi: 10.1186/1471-2474-15-54](https://doi.org/10.1186/1471-2474-15-54)
46. Steckelberg A, Hülfenhaus C, Haastert B, Mühlhauser I. Effect of evidence based risk information on "informed choice" in colorectal cancer screening: randomised controlled trial. BMJ. 2011 Jun 2;342:d3193. doi: 10.1136/bmj.d3193. PMID: 21636633; PMCID: PMC3106362.
47. Taylor KL, Davis JL 3rd, Turner RO, Johnson L, Schwartz MD, Kerner JF, et al. Educating African American men about the prostate cancer screening dilemma: a randomized intervention. Cancer Epidemiol Biomarkers Prev. 2006 Nov;15(11):2179-88. doi: 10.1158/1055-9965.EPI-05-0417. PMID: 17119044.
48. van Peperstraten A, Nelen W, Grol R, Zielhuis G, Adang E, Stalmeier P, et al. The effect of a multifaceted empowerment strategy on decision making about the number of embryos transferred in in vitro fertilisation: randomised controlled trial. BMJ. 2010 Sep 30;341:c2501. doi: 10.1136/bmj.c2501. PMID: 20884700; PMCID: PMC2948112.
49. Williams RM, Davis KM, Luta G, Edmond SN, Dorfman CS, Schwartz MD, et al. Fostering informed decisions: a randomized controlled trial assessing the impact of a decision aid among men registered to undergo mass screening for prostate cancer. Patient Educ Couns. 2013 Jun;91(3):329-36. doi: 10.1016/j.pec.2012.12.013. Epub 2013 Jan 26. PMID: 23357414; PMCID: PMC3727283.

**2019 IPDAS Establishing Effectiveness Chapter Update REDCap Codebook**

Note: Data abstraction tool repeated the following variables to include up to 12 total instruments per published paper.

| **Variable / Field Name** | **Field Label**  ***Field Note*** | **Field Attributes (Field Type, Validation, Choices, Calculations, etc.)** |
| --- | --- | --- |
| coder_initial | Coder Initial | [free text] |
| name_inst | Section Header: *List of instruments. ONLY include measures that address one or more of the key outcomes. [author_year] [paper_title]*  Instrument #1 Name  (edit below only if needed) | [free text] |
| describe_inst | Instrument #1 description (please provide details for items). Include #items, format of items, (T/F, open ended, Likert etc), subscales used (if named). | [free text] |
| dquality_header | 1. Decision Quality Outcome | *header* |
| dq_informed | Informed | \| 1 \| Yes \| \| --- \| --- \| \| 0 \| No \| |
| dq_realexpec | Realistic expectations | \| 1 \| Yes \| \| --- \| --- \| \| 0 \| No \| |
| dq_concr | Concordance | \| 1 \| Yes \| \| --- \| --- \| \| 0 \| No \| |
| dq_pref | Preference | \| 1 \| Yes \| \| --- \| --- \| \| 0 \| No \| |
| dq_choice | Choice | \| 1 \| Yes \| \| --- \| --- \| \| 0 \| No \| |
| dprocess_header | 2. Decision Process Measures  (If survey gets at any of these issues then it can be considered to be a "process" measure) | *header* |
| decprocess_recdec | Recognize decision | \| 1 \| Yes \| \| --- \| --- \| \| 0 \| No \| |
| decprocess_understand | Feel that understand options and outcomes | \| 1 \| Yes \| \| --- \| --- \| \| 0 \| No \| |
| decprocess_matters | Feel clear about what matters most | \| 1 \| Yes \| \| --- \| --- \| \| 0 \| No \| |
| decprocess_goals | Discuss goals with providers | \| 1 \| Yes \| \| --- \| --- \| \| 0 \| No \| |
| decprocess_involved | Involved in decision making | \| 1 \| Yes \| \| --- \| --- \| \| 0 \| No \| |
| othercon | Other construct: this instrument is neither decision process nor decision quality | \| 1 \| Yes \| \| --- \| --- \| \| 0 \| No \| |
| addl_papers | 3. Additional studies that used instrument and/or validation reference that is/are cited in this paper. | \| 1 \| Yes, cited \| \| --- \| --- \| \| 0 \| No, not cited \| |
| decision_type | 4. Medical Topic/Decision | [free text] |
| admin_time_measure | 5. Administration and timing  Mode of administration (paper, phone, computer, interviewer, etc.) and timing of administration | *header* |
| inst_new | 6. New Instrument? | \| 1 \| instrument developed for this study \| \| --- \| --- \| \| 2 \| instrument previously published (and/or adapted from published) \| \| 3 \| unclear \| |
| inst_quality | 7. Quality of instrument reported in this paper? | \| 1 \| Yes \| \| --- \| --- \| \| 0 \| No \| |
| notes_comments | 8. Notes and Comments | [free text] |
| develop_header | Development Process | *header* |
| develop_item | 9. Development Process (was any information on the item development included in the paper?)  Examples: content, cognitive interviews, pilot testing, etc. | \| 1 \| Reported \| \| --- \| --- \| \| 2 \| Nothing reported, but citation provided \| \| 3 \| Nothing reported \| |
| develop_itemdescription | Report item generation  Was there information about how the content of the items was developed and by whom? | \| 1 \| Yes reported \| \| --- \| --- \| \| 0 \| Not reported \| |
| develop_coginterview | Report cognitive interviews  Was the measure tested for understandability before use? | \| 1 \| Yes reported \| \| --- \| --- \| \| 0 \| Not reported \| |
| develop_pilot | Report pilot testing  Were pilot studies (of any type) conducted to pretest the measure? | \| 1 \| Yes reported \| \| --- \| --- \| \| 0 \| Not reported \| |
| report_reliability | 10. Reliability:  The instrument is reproducible and when appropriate internally consistent.  Examples of reliability testing: (1) internal consistency reliability (e.g., Cronbach's alpha, Kuder-Richardson coefficient); (2) test-retest reliability; (3) interrater reliability (e.g., percentage agreement, kappa coefficient, intraclass correlation coefficient)  Reported? | \| 1 \| Yes \| \| --- \| --- \| \| 0 \| No \| |
| describe_reliability | Describe type and result (Cronbach alpha, retest reliability, intraclass correlation coefficient, interrater reliability, etc.) | [free text] |
| report_validity | 11. Validity:  The extent to which the instrument assesses what is intended.  Examples of validity tests: (1) content validity (e.g. Content Validity Index); (2) criterion-related validity (e.g. correlations to demonstrate con-current, predictive validity); (3) construct validity (e.g. factor analysis to demonstrate predicted convergence/divergence of constructs and/or structure invariance of the measure, discriminant analysis, known groups analysis).  Reported? | \| 1 \| Yes \| \| --- \| --- \| \| 0 \| No \| |
| describe_validity | Describe type of validity test (Content Validity Index, correlations, predictive validity, discriminant analysis, etc.) | [free text] |
| report_responsiveness | 12. Responsiveness:  The instrument is sensitive to changes of importance to patients and clinicians.  Examples: NA  Reported? | \| 1 \| Yes \| \| --- \| --- \| \| 0 \| No \| |
| describe_responsiveness | Describe type of analysis and result | [free text] |
| report_precision | 13. Accuracy & Precision:  What is known about the measure performance in comparison to gold-standard measures (accuracy) and /or the number of distinctions and extent of random error in use of the measure (precision)?  Examples: NA  Reported? | \| 1 \| Yes \| \| --- \| --- \| \| 0 \| No \| |
| describe_precision | Describe type of analysis and result | [free text] |
| report_interprete | 14. Interpretability:  Evidence the scores are meaningful to clinicians and patients.  Examples: NA  Reported? | \| 1 \| Yes \| \| --- \| --- \| \| 0 \| No \| |
| describe_interprete | Describe type of analysis and result | [free text] |
| report_accept | 15. Acceptability:  Are there any indicators of acceptability to respondents (usually patients, could include others)? Are there patterns to missing data or low response rates that could signal a problem with acceptability of the measure?  Examples: (1) missing data patterns; (2) low response rate patterns NOTE: overall study response rates is not sufficient.  Reported? | \| 1 \| Yes \| \| --- \| --- \| \| 0 \| No \| |
| describe_accept | Describe type of analysis and result | [free text] |
| report_feasibility | 16. Feasibility:  Are there indicators of the appropriateness of effort, burden, or disruption (of clinical or research team) required to administer the measure?  Examples: NA  Reported? | \| 1 \| Yes \| \| --- \| --- \| \| 0 \| No \| |
| describe_feasibility | Describe type of analysis and result | [free text] |
| comms_ques_inst | Comments or additional questions | [free text] |
